# Scheme optimization of acupoints compatibility and influence factors of the effect

**DOI:** 10.1097/MD.0000000000027883

**Published:** 2021-12-17

**Authors:** Lijuan Ha, Xiaona Liu, Yanze Liu, Mujun Zhi, Hailin Jiang, Jingying Zhao, Yiming Wang, Xiaoru Xu, Le Guo, Jiazhen Cao, Liyuan Chen, Yue Yuan, Tie Li, Fuchun Wang

**Affiliations:** Department of Acupuncture, School of Acupuncture and Massage, Changchun University of Chinese Medicine, Changchun, People's Republic of China.

**Keywords:** Acupuncture, Primary insomnia, RCT

## Abstract

**Background:**

Acupuncture may be a clinically effective treatment for insomnia. We will perform a multicenter, large-scale, single-blinded, randomized controlled trial to compare the differences in the clinical efficacy between the use of singleacupoints and compatibilityacupoints in the treatment of primary insomnia.

**Methods/Design:**

A total of 333 participants will be randomly assigned to 2 acupoint treatment groups or 1 nonacupoint control group in a 1:1:1 ratio by a central stochastic system. The acupuncture groups are: the single acupoint group: Shenmen (HT7); and he compatibility acupoint group: Shenmen (HT7), Baihui (DU20), and Sanyinjiao (SP6). The observation period of this trial will be 10 weeks. All patients will be followed for 1 week before randomization (baseline phase). After randomization, the patients will receive 30 minutes of electro-acupuncture once per day for 5 weeks. In the fourth week after the treatment, follow-up will be performed once. The primary outcome will be the Pittsburgh sleep quality index score at 1 week before randomization and 2 and 8 weeks after randomization. The secondary outcomes will include data from sleep diaries, Athens insomnia scale scores, ShortForm-36 Health Survey scores, electroencephalogram technology results and polysomnogram) results. Patients will be required to complete a sleep diary every day during the treatment period. Patients will also undergo electroencephalogram technology and polysomnogram 1 week before randomization and 5 weeks after randomization. The other secondary outcomes will be measured 1 week before randomization and 5 and 9 weeks after randomization.

**Discussion:**

This trial will be helpful in identifying whether acupuncture at compatibility acupoints is more effective than acupuncture at single acupoints.

**Trial registration:**

Clinical Trials.govNCT02448602, Registered 5May 2015, https://www.clinicaltrials.gov/ct2/show/NCT02448602?term=NCT02448602&rank=1

## 1 Introduction

Insomnia is characterized by dissatisfaction with sleep quantity or quality and is associated with difficulty falling asleep and maintaining sleep.^[1]^ Most reports suggest that the prevalence rates of insomnia are 5% to 15%.^[2–5]^ Insomnia is a chronic problemand has been associated with erectile dysfunction,^[6]^ burning mouth syndrome,^[7]^ depression, ^[8]^anxiety,^[9]^ and functional gastrointestinal disorders.^[10]^ There are many ways to treat or relieve insomnia, including the “guided fantasy” technique,^[11]^ operation of a homeostatic sleep switch,^[12]^ and cognitive behavioral treatments.^[13]^ Among the dominant disciplines in traditional Chinese medicine, acupuncture and moxibustion are widely applied and promoted in clinical practice due to their safety and efficacy. Many Chinese and foreign studies have demonstrated that acupuncture has hypnotic effects and is effective in treating insomnia.^[14]^ However, questions related to the selection of acupoints to treat insomnia, as well as whether single acupoints or compatibility acupoints have better therapeutic effects, have affected clinical acupoint treatment, and these questions remain open.

Therefore, we will perform this multicenter, randomized, controlled, large-scale trial, first aiming to investigate whether acupuncture is effective in treating insomnia through a comparison with a non-acupoint control group. Second, we will compare the clinical efficacy of single acupoints with that of compatibility acupoints in the treatment of primary insomnia. The work reported in this article is financed by the National Basic Research Program (973 Program: 2014CB543100) in China and is registered with an identifier (NCT02448602) by Clinical Trials.gov in the United States of America.

## 2 Methods

### 2.1 Design

This study will be a multicenter, large-sample, randomized, controlled trial conducted in the following 6 hospitals: Affiliated Hospital of Changchun University of Traditional Chinese Medicine; The First Affiliated Hospital of Hunan University of Traditional Chinese Medicine; Hengyang Affiliated Hospital of Traditional Chinese Medicine; People's Affiliated Hospital of Traditional Chinese Medicine; China-Japan Friendship Hospital of Jilin University; and QiLu Hospital of Shandong University. The design of this study is in accordance with the guidelines of the HIS Committee on Clinical Trials in Migraine.^[15]^

**Figure 1:**
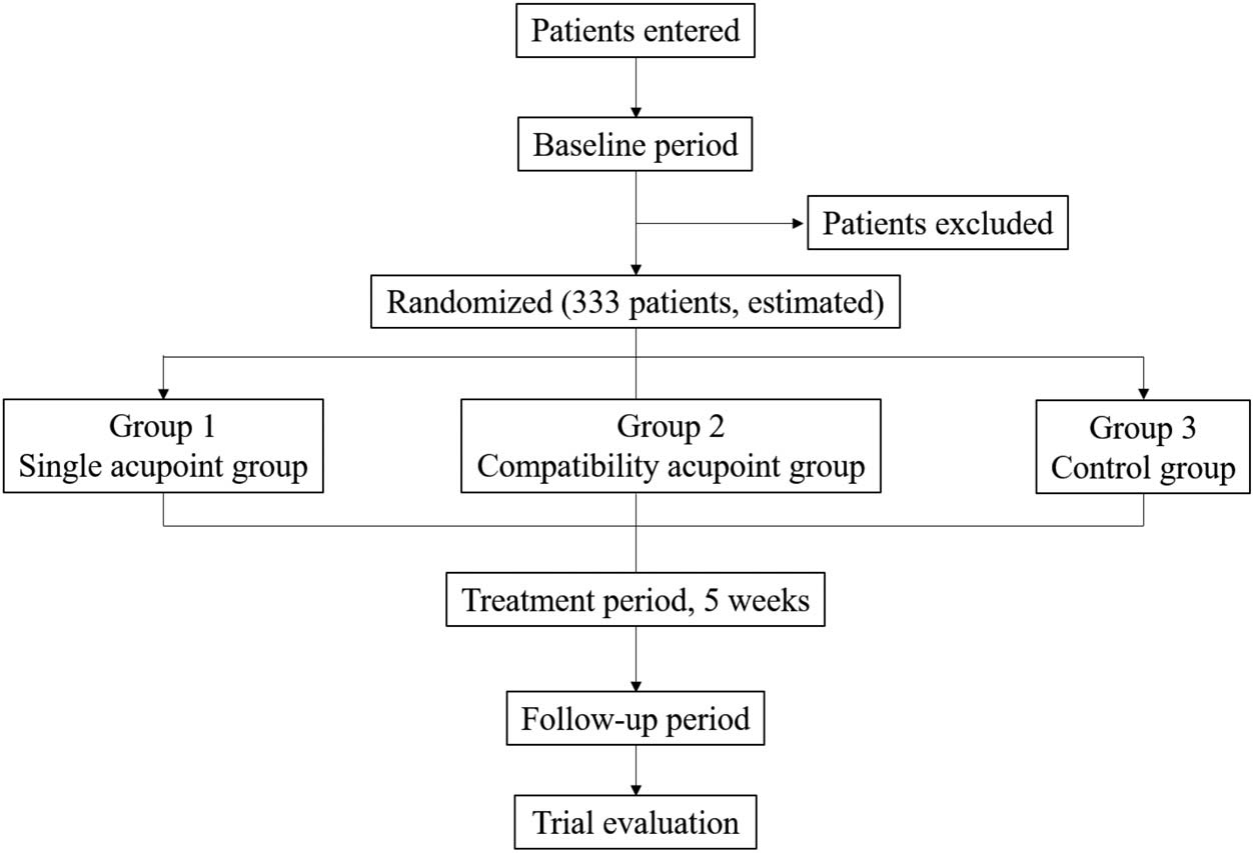
Trial flow chart.

This trial will include 3 groups (Fig. 1, Fig. 2 ): 2 acupoint treatment groups (single acupoint and compatibility acupoint groups) and 1 nonacupoint control group. A central stochastic system will be used to distribute the patients among the 3 groups equally.

**Figure 2:**
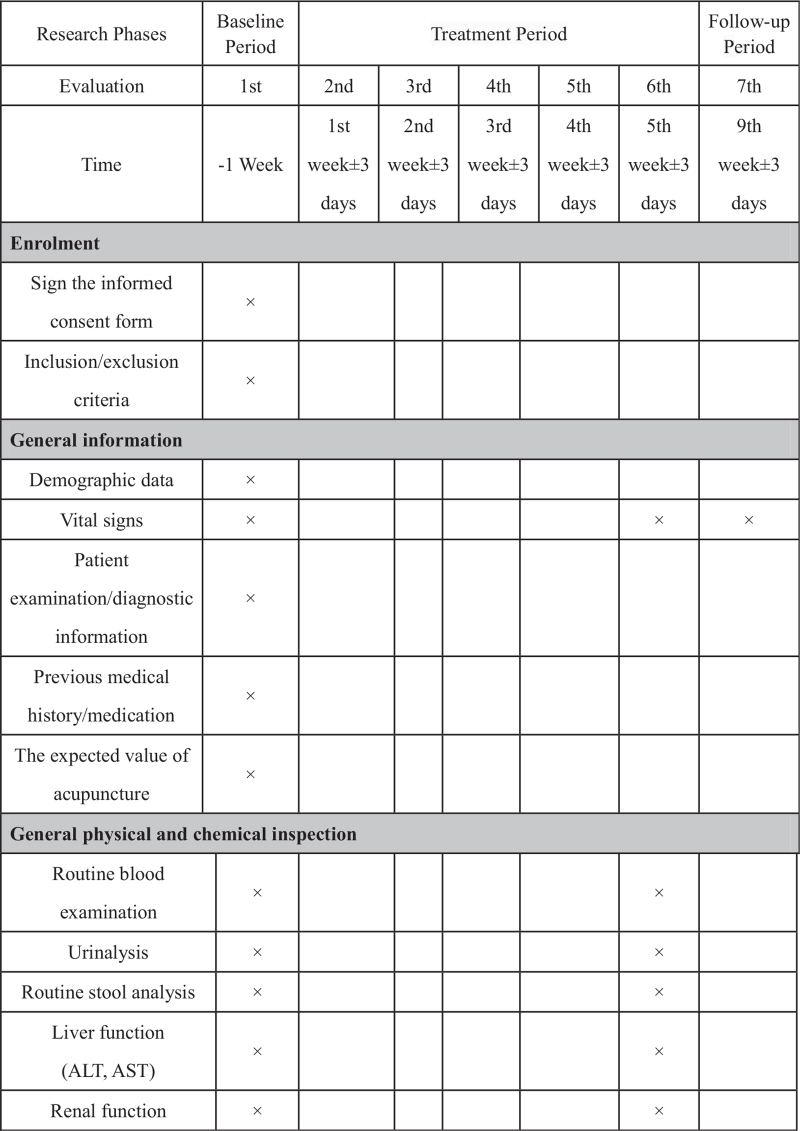
Trial process chart.

**Figure 2 (Continued):**
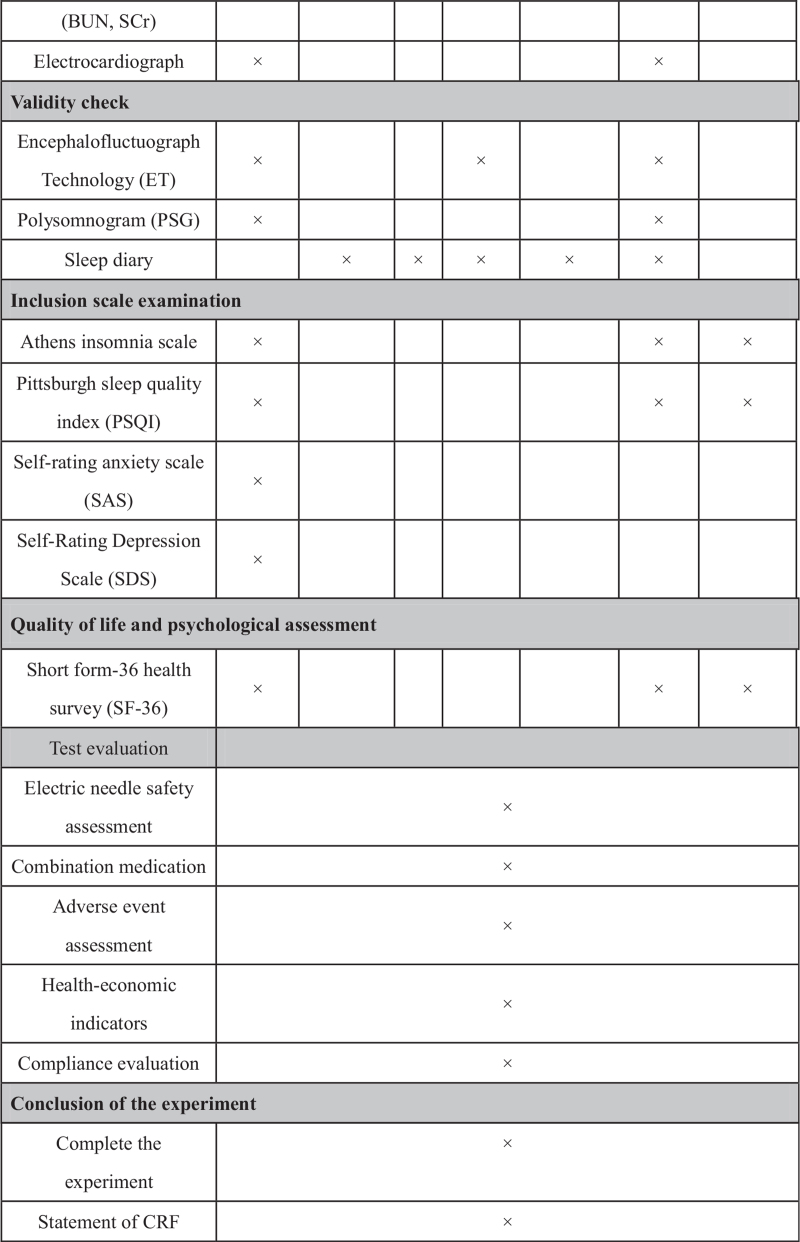
Trial process chart.

The total observation period of this trial will be 10 weeks. All patients will be followed for 1 week before randomization (baseline phase). After randomization, the patients will receive 30 minutes of electroacupuncture once per day for 5 weeks. In the fourth week after the treatment, follow-up will be performed 1 time.

The single-blind evaluation method will be used in this trial. Nevertheless, the patients will be informed that they will be in either one of the acupoint treatment groups or the nonacupoint control group, which is the most effective way to avoid bias.

To communicate in a timely manner between each subcenter and to improve the efficiency of the trial, central randomization will be used, as performed by the China Academy of Traditional Chinese Medicine clinical evaluation center. The patients will be registered in the groups by phone or network at any time and any geographic location, such that the test manager can monitor the progress of each subcenter subject in real time.

This trial will be performed according to the principles of the Declaration of Helsinki (Version Edinburgh 2000). The trial protocol has been approved by the local institutional review board and ethics committee. Written informed consent will be requested from all participants before registration. All patients will be given enough time to decide whether they should sign the informed consent form, and they will be given treatment options other than acupuncture if they are not willing to participate.

### 2.2 Patients

#### 2.2.1 Diagnostic criteria

According to the American Psychiatric Association (APA) Diagnostic and Statistical Manual of Mental Disorders –DSM-5 (2013) diagnostic criteria for insomnia, the diagnosis of insomnia must meet the following conditions:

1. The individual reports ≥1 of the following sleep-related complaints: difficulty initiating sleep, difficulty maintaining sleep, waking up too early, or sleep that is chronically non-restorative or poor in quality.
2. The sleep disturbance (or associated daytime fatigue) causes clinically significant distress or impairment in social, occupational or other important areas of functioning.
3. The sleep disturbance has occurred at least 3 times per week.
4. The complaint has lasted at least three months.
5. The disturbance is not due to the direct physiological effects of a substance.

#### 2.2.2 Inclusion criteria

1. Meets the diagnostic criteria for insomnia.
2. Is between 18 and 60 years’ old.
3. Is not participating in other clinical trials.
4. Is willing to finish the treatments and has signed the consent form.
5. Has a PSQI index >7.
6. Has an Athens index ≥6.
7. Has depression scale and anxiety scale scores ≤50.

#### 2.2.3 Exclusion criteria

Patients with any of the following conditions will be excluded:

1. Respiratory-related sleep disorders or circadian rhythm sleep disorders.
2. Insomnia caused by drugs (eg, drug abuse, drug therapy) or sleep disorders caused by excessive consumption of alcohol, coffee or strong tea, or other lifestyle factors.
3. Insomnia caused by the direct physiological effects of a substance.
4. Severe primary diseases involving the cardiovascular, lung, liver, kidney or hematopoietic systems, or other serious primary diseases.
5. Pregnancy or lactation.
6. Allergic constitutions or susceptibility to infections or bleeding.
7. Poor compliance.
8. Psychosis.
9. Insomnia caused by excessive anxiety, depression, or other causes.

### 2.3 Elimination and shedding standards

1. Cases that meet the exclusion criteria.
2. Those who fail to comply with the prescribed treatment or have incomplete data affecting the evaluation of efficacy and safety should be excluded.
3. Subjects with poor compliance who quit on their own during the course of treatment.
4. Combined use of therapeutic methods that are prohibited under this programme or replacement of treatment methods on their own.
5. If serious adverse events or complications occur, and it is not appropriate to continue the treatment; treatment should be discontinued.

### 2.4 The treatment of removed cases

1. When a subject drops out, the chief physician should employ methods such as going to the subject's house, making phone calls, or sending letters to try to contact the subject to ask for the reasons for study dropout, to record the last date of treatment, and to complete the study analysis.
2. The chief physician should use alternate treatment measures according to the actual situation of the subjects who are no longer in the study because of adverse reactions or treatment ineffectiveness.
3. When a subject is assigned a random number, he/she becomes an object of the experimental observation, regardless of future diagnosis or the integrity of the treatment.
4. After completing at least half the treatment course, a subject who drops out will be considered an effective evaluation case; less than half of the patients with adverse reactions will not be evaluated and will be evaluated only for adverse reactions. However, patients who violate the test plan (eg, violation of the combination of drugs, violation of the inclusion criteria, and medication compliance) will not be evaluated for efficacy or adverse reactions.

#### 2.4.1 2.2.6 Management of Adverse Events

1. Records and reports
  The investigator should explain to the patient that they (or a family member) should accurately report all physical changes after treatment. The doctor should avoid leading questions and observe the adverse reactions when observing the effects. Whether adverse effects or adverse events are associated with the treatment methods in this study, they should be detailed in the record, including adverse reaction times, symptoms, physical signs, adverse reaction degrees, adverse reaction durations, laboratory examination indices, treatment methods, and results and follow-up times.
  If serious adverse events occur during the course of treatment, the investigator must report the adverse events to the clinical research department within 20 hours or no later than the second working day. The investigator will sign and date the report. The investigator should record when, how and to whom they reported the serious adverse events in the original data.
2. Treatment of patients
  In cases of adverse events, the investigator may take necessary measures according to the condition of the disease until the situation is stable. Patients with abnormal test results should be tracked until the results return to normal. The investigator will decide whether to end the observation. The follow-up method can be selected according to the degree of adverse events, including hospitalization, outpatient observation, home visit, telephone call, and communication.

### 2.5 3 Interventions

#### 2.5.1 Grouping and acupoint selection

1. Single acupoint group: Shenmen (HT7).
2. Compatibility acupoint group: Shenmen (HT7), Baihui (DU20), Sanyinjiao (SP6).
3. Control group: nonacupoint.

#### 2.5.2 Acupoint locations and treatment

The locations of points in the single acupoint and compatibility acupoint groups will be referenced using the national standard of the People's Republic of China in 2006 (GB/T 12346–2006) in the following form: “The name and location of Acupoint ." The acupuncture will be applied based on “Acupuncture and Moxibustion,” edited by Liang Guangsheng, the Ministry of Health's 12th five-year plan.

Shenmen (HT7) is located on the anteromedial aspect of the wrist, radial to the flexor carpi ulnaris tendon, on the palmar crease and should be punctured perpendicularly 0.3 to 0.5 cun. Sanyinjiao (SP6) is on the tibial aspect of the leg, posterior to the medial border of the tibia, and 3 cun superior to the prominence of the medial malleolus, and it should be punctured perpendicularly 1.0 to 1.5 cun. Baihui (DU20) is located on the head, 5 cun directly above the midpoint of the anterior hairline, at the midpoint of the line connecting the apexes of both ears, and it should be punctured transversely 0.5 to 1.0 cun. The nonacupoint is in the medial arm on the anterior border of the insertion of the deltoid muscle at the junction of the deltoid and biceps muscles and is punctured perpendicularly 2 mm.

HT7, SP6, and DU20 require lifting and thrusting, combined with twirling and rotating, of the needles to achieve the DeQi sensation, and the needles will be retained for 30 minutes. The nonacupoint patients will not be asked about the DeQi sensation or any manipulation.

#### 2.5.3 Needles

The needles are 25 mm in length and 0.25 mm in diameter or50 mm in length and 0.30 mm in diameter. The needles that are 13 mm in length and 0.18 mm in diameter will be used as auxiliary needles and will not be manipulated.

The sterile acupuncture needles used in this trial, namely, Hwato needles, made by Suzhou Medical Supplies Factory in China, are for single use.

### 2.6 Operating physician

The physicians who will participate in this trial will be required to pass training in standard clinical operating procedures.

SOP for the basic operation of acupuncture:

1. Disinfection
  Disinfection of the doctor's hands: Before acupuncture, the doctors will wash their hands with soap and water and then wipe their hands with 75% alcohol swabs before holding the needles.
  Disinfection of the acupuncture site: The local skin at the acupuncture site will be wiped and sterilized with 75% alcohol cotton balls, and a ring will be sterilized from the center to the surroundings.
2. Needling
  Using the 2-handed approach, according to the characteristics of the location of the acupuncture points, which will be used to select the claw cutting or clamping method, the direction and depth of acupuncture will be in strict accordance with the requirements of acupuncture operation.
3. Retention of the needles
  We expect that the sham acupuncture points and the needle points will be plugged and twirled, and “deqi”will be performed simultaneously. All needles will be left in place for 30 minutes and will not be removed during the needle retention process.
4. Needle removal
  With the left thumb and index finger holding the sterile dry cotton ball gently pressed on the acupuncture site, holding the needle in the right hand for slight twisting, the practitioner will slowly bring the needle to the skin, let it rest for a moment, and then take the needle out, pressing the pinhole to prevent bleeding.
5. The electroacupuncture operation
  After each acupoint or nonacupoint is needled, auxiliary needles will be used for puncture at locations 2 mm lateral to every acupoint or nonacupoint to a depth of 2 mm without manual stimulation. After needle insertion, transcutaneous electric acupoint stimulation (HAMS: Han's acupoint nerve stimulator, HANS-200, Nanjing, China) will be used for electroacupuncture stimulation at every acupoint or nonacupoint.
  Each acupuncture needle used for acupoints or nonacupoints and their auxiliary needles will be connected to electricity by the HANS for 30 minutes. The stimulation frequency will be 60/100 Hz, and the stimulation intensity will vary from 0.1 to 1.0 mA until the patients feel comfortable.
6. Treatment course
  All patients will receive 25 treatments over a period of 5 weeks, once a day, 5 times consecutively with 2-day breaks every week. Follow-up will be performed in the fourth week after treatment.

### 2.7 Statistical analysis

#### 2.7.1 Outcome measurement

The primary outcome measure in this trial will be the PSQI, and the secondary outcome measures will include the sleep diary, Athens insomnia scale score, SF-36 score, ET results, and PSG results.

PSQI^[16,17]^ is a self-rated questionnaire that assesses sleep quality and disturbances over a 1-month time period. Nineteen individual items generate 7 “component” scores: subjective sleep quality, sleep latency, sleep duration, habitual sleep efficiency, sleep disturbances, use of sleeping medication, and daytime dysfunction. The sum of scores for these 7 components yields 1 global score. All patients will complete the PSQI 1 week before randomization and in the fifth and eighth weeks after randomization.

The sleep diary^[18]^ will be used to record the patients’ sleep and to collect sleep information to assess the therapeutic effect after treatment. Patients will need to complete the sleep diary every day during the treatment period and complete the CRFs only once a week.

The Athens insomnia scale will be used to record the patients’ self-assessment of sleep disorders. The items include sleep latency, waking up at night, waking up earlier than expected, total sleep time, sleep quality, emotions during the day, physical functioning during the day and daytime sleepiness. Patients will need to complete the Athens insomnia scale 1 week before randomization and at the fifth and the ninth week after randomization.

The SF-36^[19]^ questionnaire consists of 36 items containing 8 subscales each. These subscales consist of 2 higher-level summary scales, namely, the physical (physical function, physical role function, bodily pain, and general health) and psychological (vitality, social function, emotional role function, and mental health) summary scales. Patients will complete the SF-36 1 week before randomization and at the fifth and ninth week after randomization.

ET is a pioneering analysis technology in China that provides noninvasive, quantitative detection of the neurotransmitters involved in brain activity and brain function regardless of physiological or pathological condition. It can be applied to the study of brain function, providing objective mathematical evidence for the study of neuropsychiatric diseases and clinical treatment guidance. Patients will need to perform ET 1 week before randomization and at the third and fifth weeks after randomization. Patients will also need to undergo PSG 1 week before randomization and at the fifth week after randomization.

Biological indicators, including body temperature, heart rate, respiration and blood pressure, will be measured 1 week before randomization and at the fifth and ninth weeks after randomization. Sex, age, culture, occupation, height, weight, and acupuncture expectation will also need to be recorded.

Any adverse events and the manner in which they are addressed will be recorded during the 4 treatment weeks and the 12 follow-up weeks. These adverse events include bleeding, hematoma, fainting, serious pain, and local infection. If patients suffer any serious adverse events, all details will be documented. The patients’ blood, urine, stool, liver function, renal function, and electrocardiogram analysis results will be recorded 1 week before randomization and at the fifth week after randomization.

Drop-outs, withdrawals, and patient compliance will be recorded during the treatment weeks and the 4 follow-up weeks. All patients will be recorded until the end of the trial.

### 2.8 Sample size

A power analysis is calculated before conducting this trial, which α = 0.05 and power = 90%. According to the previous pilot study, percentages of responders for the primary endpoint are verum 87.5%, sham 25%. Therefore, the sample size in this trial can be estimated initially according to the following formula. Each group needs at least 111 patients (assuming a 20% dropout rate), 333 patients in 3 groups totally will be enrolled in the study.


$$n=\frac{2{\left(u_{\alpha }^{'}+u_{\beta }\right)}^{2}p(1-p)}{{\left(p_{1}-p_{2}\right)}^{2}}$$

### 2.9 Statistical analysis

#### 2.9.1 Validity analysis

The efficacy scores for week 0, week 2, and week 4 for patients with primary insomnia will be calculated using the difference between the treatment period and baseline period. These indicators will be described by mean, standard deviation, median, P5, P75, and maximum and minimum values. PP and ITT will be used to analyze the main indicators and overall indicators. Because this study is a multicenter randomized controlled clinical trial, the central effect on the therapeutic effect will be considered in the analysis.

#### 2.9.2 Effect factor analysis

If there are obvious differences between the groups in age, gender, disease type or condition before the test or there are factors that will significantly alter the curative effect, the analysts will need to consider these factors as covariates when comparing the groups. Covariance analysis or logistic regression analysis will be used, and the combination of drugs and other conditions will be listed in detail.

#### 2.9.3 Safety analysis

Depending on the requirements of adverse reactions related to the control and treatment groups, adverse events and adverse reactions (the number of cases and the probability of occurrence) will be listed in a form, and the reasons for their occurrence will be described. If necessary, the *χ*^2^ test can be used for the statistical analysis of adverse reactions.

Statistical analysis will be performed using SAS9.1.3 statistical analysis software. *P* < .05 (α = 0.05) will be considered to be statistically significant.

## 3 Discussion

This trial is a portion of the National Basic Research Project (973 Program, 2014CB543100). The 973 Program, the national key basic research development plan, aims to solve major scientific problems associated with national strategic needs and advance the frontiers of science that will play an important role in understanding the world.

Because acupuncture works quickly and is easy to perform, it has greater application and popularization value clinically. Acupuncture was listed in the “representative list of intangible cultural heritage of humanity” and is widely used in >160 countries and regions in the world. The normative selection of acupoints in clinical practice is a major scientific problem, as is determining the development of acupuncture theory and the improvement of clinical efficacy. It is also the historical mission of modern acupuncture practitioners.

The patterns of acupoint compatibility are many and varied in clinical practice. The basic pattern is compatibility according to the location and meridian; however, each acupoint has a specific therapeutic effect, and only the appropriate compatibility will achieve the best effect. Therefore, identification of scientific and reasonable acupoint compatibility is the key to improving the effectiveness of acupuncture.

Generations of physicians have agreed that acupoint compatibility can enhance the curative effect, and many propose the compatibility method. Some achievements have been made in the study of the compatibility of modern methods. There are 874 related studies and reviews worldwide. However, there is no scientific basis regarding whether the compatibility acupoints are superior to a single acupoint, and there is a lack of evaluation systems of the variable effect between various acupoints and the optimal compatibility formula. The key factors that influence the effect of compatibility are also not clarified. Therefore, the main goals of compatibility acupoint research are definition of the optimal formula of compatibility, evaluation of the methodology, and explanation of the influencing factors of the various effects of compatibility.

For this trial, we chose insomnia as an outcome measure, guided by traditional Chinese medicine and acupuncture, combined with acupuncture clinical research standards, clinical epidemiology, and evidence-based medicine. We adopted a multicenter, maximal sample randomized controlled trial approach. By means of objective and scientific evaluation, we will compare the various effects of compatibility acupoints.

## Author contributions

**Conceptualization**: Lijuan Ha, Xiaona Liu

**Data curation**: Lijuan Ha, Xiaona Liu, Yanze Liu

**Formal analysis**: Hailin Jiang, Xiaoru Xu

Funding acquisition: Fuchun Wang

**Methodology**: Yanze Liu, Yiming Wang

Project administration: Fuchun Wang, Le Guo

**Resources**: Fuchun Wang, Liyuan Chen

**Software**: Yanze Liu, Mujun Zhi, Yiming Wang

**Supervision**: Hailin Jiang, Jingying Zhao

**Validation**: Mujun Zhi, Jingying Zhao, Yue Yuan

**Visualization**: Fuchun Wang, Jiazhen Cao

**Writing – original draft**: Lijuan Ha, Xiaona Liu

Writing – review & editing: Lijuan Ha

**Conceptualization:** Lijuan Ha, Xiaona Liu, Tie Li.

**Data curation:** Lijuan Ha, Xiaona Liu, Yanze Liu.

**Formal analysis:** Hailin Jiang, Xiaoru Xu.

**Funding acquisition:** Fuchun Wang.

**Methodology:** Yanze Liu, Yiming Wang.

**Project administration:** Le Guo.

**Resources:** Liyuan Chen, Fuchun Wang.

**Software:** Yanze Liu, Mujun Zhi, Yiming Wang.

**Supervision:** Hailin Jiang, Jingying Zhao.

**Validation:** Mujun Zhi, Jingying Zhao, Yue Yuan.

**Visualization:** Jiazhen Cao, Fuchun Wang.

**Writing - original draft:** Lijuan Ha, Xiaona Liu.

**Writing - review & editing:** Lijuan Ha, Tie Li.
